# The Effects of Uncomplicated Cataract Surgery on Retinal Layer Thickness

**DOI:** 10.1155/2018/7218639

**Published:** 2018-06-05

**Authors:** Ali Kurt, Raşit Kılıç

**Affiliations:** Department of Ophthalmology, Faculty of Medicine, Ahi Evran University, Kırşehir, Turkey

## Abstract

**Purpose:**

Our aim was to assess changes in the total retinal thickness (TRT), total retinal volume (TRV), and retinal layer thickness after uncomplicated cataract surgery.

**Methods:**

A total of 32 eyes of 32 patients who had undergone uncomplicated phacoemulsification surgery and intraocular lens implantation in one eye were enrolled. Effective phacoemulsification time (EPT) and total energy (TE) were recorded. Thickness and TRV were measured using optical coherence tomography. Data were collected preoperatively and at postoperative day 1, 7, 30, 90, and 180.

**Results:**

The study results showed a decrease in TRT, TRV, and most retinal layer thicknesses at the first postoperative day visit and then increasing at week 1, and months 1 and 3, and then relatively decreasing at month 6 although not returning to preoperative levels. The least affected layers were the retinal pigment epithelium and outer plexiform layer. There was a positive correlation between EPT and TE and ganglion cell layer in a 1 mm circle and inner nuclear layer in a 1–3 mm circle (*p* < 0.05).

**Conclusion:**

The results suggest that long-term follow-up of more than 6 months is necessary after cataract surgery to see whether total retinal and segmental values return to preoperative levels. This study was registered with Australian New Zealand Clinical Trials Registry (ANZCTR): ACTRN12618000763246.

## 1. Introduction

Cataract is the most common preventable cause of vision loss worldwide. Pseudophakic cystoid macular edema (PCME), known as Irvine–Gass syndrome, is one of the most common complications after cataract surgery. It is generally subclinical in most cases and rarely causes vision loss. Although the incidence of clinical PCME has decreased with small incision cataract surgery and phacoemulsification (PE), it can still cause unexpected vision loss [1]. The exact pathophysiology of PCME is not fully understood but seems to be related to the inflammation triggered by surgery. The inflammatory cytokines and mediators break down the blood-retina barrier and result in increased vascular permeability and cystoid macular edema [2, 3]. Other factors such as posterior capsule rupture, vitreous loss, retained lens fragments, vitreomacular traction, and iris trauma after complicated surgery can also increase the PCME incidence [1–3].

PCME is most commonly seen 4–6 weeks after surgery [1–3]. Fundus fluorescein angiography (FFA) reveals capillary dilatation, leakage from the foveal capillaries, and developing petalloid appearance. Optical coherence tomography (OCT) is a noninvasive device which enables detection of cystic spaces, retinal thickening, and subretinal fluid. OCT also has good repeatability and reproducibility when measuring retinal layer thickness at the macula [4]. It is an excellent method for monitoring disease activity [3].

The current knowledge on the effect of postoperative inflammation on retinal cells and layers is limited. We are not aware of any study assessing the retinal segments to detect the layers that are most affected by cataract surgery with long-term follow-up. The purpose of this study was to evaluate the thickness of each retinal segment quantitatively with spectral domain (SD)-OCT before and after uncomplicated cataract surgery to gain additional information on PCME.

## 2. Methods

This prospective study was conducted at the Ahi Evran Training and Research Hospital between December 2016 and October 2017. The study was approved by the institutional review board and adhered to the tenets of the Declaration of Helsinki. Informed consent was obtained from all the patients. A total of 43 eyes of 43 Caucasian patients who had undergone uncomplicated cataract surgery and posterior chamber intraocular lens implantation were included. Eleven patients were excluded due to the lack of follow-up examinations, and the study was finally conducted on the 32 eyes of 32 patients. The visual acuity was evaluated with a Snellen chart, and a detailed biomicroscopic anterior and posterior segment examination was performed with pupillary dilatation. Air puff tonometry was used to measure the intraocular pressure. The axial length was measured using optical low-coherence reflectometry (Lenstar LS 900, Haag-Streit AG, Koeniz, Switzerland). Best corrected visual acuity was 2/20 and higher in all patients preoperatively.

Exclusion criteria consisted of macular pathologies, retinal vascular occlusion, history of any other ocular disorders (including uveitis, severe dry eye, eye trauma, glaucoma, and pseudoexfoliation syndrome) or surgery, any systemic disorders (such as diabetes, hypertension, asthma, or chronic obstructive pulmonary disease), systemic inflammation (inflammatory bowel disease and hepatitis B or C), the current use of any topical or systemic medication or anti-inflammatory agent, and intraoperative complications such as posterior capsular rupture, vitreous loss, iris prolapse, and low scan quality images due to dense cataract.

Cataract surgery was performed with the Infiniti PE device (Alcon Inc., Forth Worth, TX, USA) using a torsional handpiece. The stop and chop technique was used in all cases. Effective phaco time and phaco energy were recorded. Postoperatively, all patients were prescribed topical moxifloxacin and dexamethasone four times a day for three weeks and Nevanac three times a day for four weeks. The same author (AK) performed all surgeries and examinations.

### 2.1. OCT Scan Protocol

**Figure 1:**
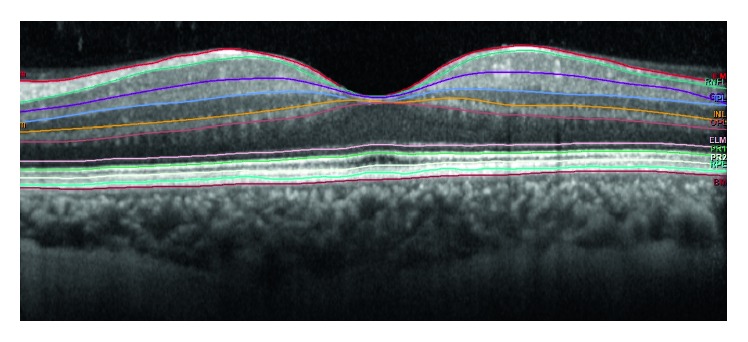
Borders of automatically segmented retinal layers on OCT images. ILM, internal limiting membrane and inner border of the RNFL layer; RNFL, outer border of the retinal nerve fiber layer; GCL, outer border of the ganglion cell layer; IPL, outer border of the inner plexiform layer; INL, outer border of the inner nuclear layer; OPL, outer border of the outer nuclear layer; ELM, external limiting membrane—outer border of the outer nuclear layer; RPE, retina pigment epithelium; BM, Bruch's membrane.

**Figure 2:**
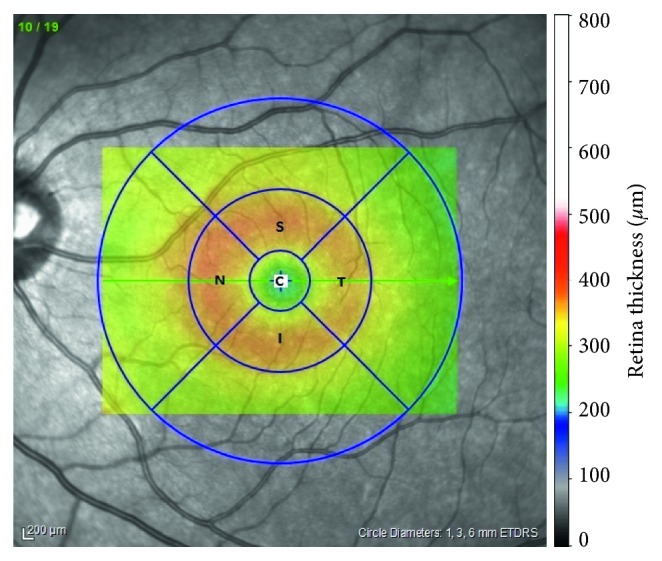
ETDRS grid for 1 mm and 1–3 mm circles on OCT images. ETDRS grid on macula. C, central 1 mm zone in macula; S, superior quadrant in 1–3 mm circle on macula; I, inferior quadrant in 1–3 mm circle on macula; T, temporal quadrant in 1–3 mm circle on macula; N, nasal quadrant in 1–3 mm circle on macula.

All subjects underwent pupillary dilatation with 1% tropicamide and 2.5% phenylephrine hydrochloride eye drops prior to imaging. We used the SD-OCT, Spectralis (Heidelberg Engineering, Heidelberg, Germany) device with software version 6.3.3.0 in this study as it has a higher repeatability index [4]. OCT imaging was carried out using the following parameters: 20° × 15° degrees (5.9 × 4.4 mm), automatic real-time averaging of 100 frames, 19 horizontal sections at 240 *µ*m intervals, and 512 A-scans per B-scan. We only included images with a quality higher than 15 dB in the study. The image acquisition was followed by automatic intraretinal layer segmentation performed by the inbuilt Spectralis software to include the retinal nerve fiber layer (RNFL), ganglion cell layer (GCL), inner plexiform layer (IPL), inner nuclear layer (INL), outer plexiform layer (OPL), outer nuclear layer (ONL), retina pigment epithelium (RPE), total retinal volume (TRV), and total retina thickness (TRT) (Figure 1). Intraretinal layer thicknesses were obtained for each ETDRS subfield at a central 1 mm circle and 1–3 mm circles that included the superior, temporal, inferior, and nasal subfields (Figure 2). The first Spectralis scan was set as a reference image, and the images during future visits were acquired with real-time image registration by follow-up mode by the ophthalmologist. The ETDRS grid was centered on the fovea manually if it was not positioned correctly automatically. We also checked the accuracy of retinal layer segmentation in every patient. The 3–6 mm subfields were not included as it exceeded the area of our imaging angle. Data were collected preoperatively and on postoperative day 1, 7 (first week), 30 (first month), 90 (third month), and 180 (sixth month). The mean thickness of the 1 mm and 1–3 mm rings was calculated and used for further statistical analysis.

### 2.2. Statistical Analysis

The IBM SPSS version 20.0 (IBM Corporation, Armonk, NY, USA) software was used for statistical analyses. Measured data were described as the arithmetic mean ± standard deviation, whereas categorical data were described as percentages (%). Normal distribution of measured data was examined by the Kolmogorov–Smirnov test. The one-way ANOVA test was used for intergroup comparison variables for repeated measures. The Bonferroni method was used to correct the *p* value. The relationship between EPT and TE and all thickness parameters were analyzed with the Pearson correlation analysis. A statistical level of significance was accepted at *p* < 0.05.

## 3. Results

The mean age of the patients consisting of 25 (78%) males and 7 (22%) females was 63.81 ± 9.0 years (range: 48–79 years). There were 20 right and 12 left eyes. The mean preoperative axial length was 23.62 ± 0.9 mm (range: 21.3–25.2 mm). The cataract type was nuclear sclerosis in 16 (50%) cases, posterior subcapsular in 12 (37.5%) cases, cortical in 3 (9.4%) cases, and cortical + posterior subcapsular in one (3.1%) case.

We found statistically significant differences in TRT and TRV in the 1 mm circle and TRT, TRV, ONL, OPL, INL, IPL GCL, and NFL in the 1–3 mm circle compared to the preoperative values during the follow-up visits continuing for 6 months (*p* < 0.05). The study results showed a remarkable decrease in TRT, TRV, and the thickness of most retinal layers at the first day visit after surgery. However, an increase was then observed in all parameters and reached approximately the preoperative values at the first week visit. The thickest TRT and retinal layer thickness values were observed at the first and third month visits. A slight decrease, not reaching the preoperative levels, was then seen in almost all parameters at the sixth month visit. We also noticed that the least affected layers were the RPE and OPL. The results are presented in Table 1.

The mean effective phacoemulsification time and total energy were 62.46 ± 45.03 seconds and 6.41 ± 7.34, respectively. There was a positive correlation between EPT and TE and GCL in the 1 mm circle and INL in the 1–3 mm circle (*p* < 0.05 and Table 2). There was no significant correlation between EPT and TE and other retinal layers, TRT and TRV (*p* > 0.05).

## 4. Discussion

The main triggering factor of PCME is thought to be surgical trauma of intraocular tissues by inducing the release of inflammatory mediators although other possible mechanisms such as photic retinopathy or vitreous traction have also been implicated [5]. Inflammatory mediators (prostaglandins, cytokines, and other vascular permeability factors) are known to be released from the anterior segment of the eye after surgery and then diffuse into the vitreous cavity and retina, stimulating the breakdown of the blood-retinal barrier (BRB) and subsequent leakage of fluids across the retinal vessel wall and into the perifoveal retinal tissues, resulting in macular edema [3]. This edema usually resolves spontaneously and only about 1–3% of cases persist, corresponding to clinical PCME with persistent symptoms [6]. Although FFA used to be considered the diagnostic gold standard for PCME, OCT is now the method of choice, being a noninvasive technique for PCME evaluation and follow-up [3].

Optical coherence tomography is a useful device to detect intraretinal cysts that indicate clinical PCME and can decrease vision noninvasively after cataract surgery [3]. Assessing the retinal layers in vivo may provide more information to elucidate the pathologic processes involved in subclinical PCME. We therefore evaluated retinal layers by OCT after uncomplicated cataract surgery and presented long-term follow-up results on TRT, TRV, and retinal layer thickness according to the ETDRS grid. We noticed that the RPE and OPL were the least affected layers. In general, we observed a decrease in TRT, TRV, and most retinal layers at the first postoperative day visit. An increase was then seen in all thickness parameters and reached approximately the preoperative levels at the first week visit. The largest TRT and retinal layer thickness values were observed at the first and third month visits. At the sixth month visit, a slight decrease was seen in almost all parameters. However, this decrease did not reach preoperative thickness levels. There was a significant thickness increase in all retinal layers except RPE and OPL in the 1–3 mm circle.

Grewing and Becker measured the retinal thickness before and 0.5 hours after cataract surgery in 10 patients and reported a decrease that was not statistically significant [7]. We noticed a decrease in TRT, TRV, and the thickness of most retinal layers after the first postoperative day. Perente et al. [8] also reported a mild postoperative retinal thickness that was not statistically significant. According to the authors, the decrease observed in the first postoperative day may be related to the previous light-scattering effect of the cataract that was possibly disrupting the optical quality of the OCT imaging [8]. However, there is not enough evidence or information in the literature to fully explain the cause.

Šiško et al. [9] reported highest retinal thickness in the ETDRS grid areas one month after uncomplicated cataract surgery. They also stated mild decreasing trend in the measurements from the first month to the sixth month, without reaching preoperative levels. Most studies have reported an increase in macular thickness after uncomplicated cataract surgery [8, 10–16]. Gharbiya et al. [10] reported a significant macular thickness increase for up to six postoperative months in 40 healthy patients. Falcão et al. [11] also found increased central macular thickness postoperatively and reported this as a nonpathological change. Cagini et al. [12] found an asymptomatic postoperative macular thickness increase at 12 weeks in 62 eyes with a follow-up period of 28 weeks. These results are all similar to ours. Gołebiewska et al. [17] reported increased retinal thickness and retinal volume during follow-up continuing for 6 months after uncomplicated cataract surgery. We observed increased retinal volume after surgery, like others.

Measuring each retinal layer separately makes it easier to see alteration in retinal structures than the TRT. It is unclear which retinal layer(s) has the most effect on increasing the retinal thickness. We found an increase in the thickness of NFL, GCL, IPL, INL, and ONL and a decrease in OPL, but these changes were only significant in the 1–3 mm circle at the postoperative sixth month follow-up when compared to the preoperative measurements. RPE thicknesses were generally stable except for the first visit, but this first-visit change was not significant. We found increased GCL thickness in the 1 mm circle and INL thickness in the 1–3 mm circle with more TE and EPT. Another study reported a statistically significant relationship between increased retinal thickness and higher perioperative phaco power [17]. However, there is no study comparing postoperative retinal layer thickness with TE and EPT values.

The INL includes the nuclei of the bipolar, horizontal, amacrine, and Muller cells. The deep capillary plexus is also in this layer. Park et al. [18] have shown that the vascular endothelial growth factor (VEGF) has a crucial role in the vitality of the amacrine and bipolar cells. Sigler et al. [19] have reported cystic changes in the INL and ONL in patients with clinical PCME. We did not find clinical PCME and therefore did not observe cystic changes in any of our patients; an increased thickness of the INL may be related to the inflammatory effects of VEGF, which is an inflammatory mediator [20]. INL thickness was also increased in relation to optic neuritis, which is an inflammatory disease, in another study [21]. In the neurology literature, the use of INL as a parameter to monitor the efficacy of anti-inflammatory treatments in multiple sclerosis has been proposed [22]. The superficial capillary plexus is located in the NFL, and its hyperpermability may have been responsible for the significantly increased thickness of the NFL and GCL in our study.

Nepafenac (Alcon Research Ltd., Fort Worth, TX, USA), a topical ocular nonsteroidal anti-inflammatory drug (NSAID) used to treat the pain and inflammation associated with cataract surgery, is available as an ophthalmic suspension in concentrations of 0.1% and 0.3% [23]. Unlike other NSAIDs, nepafenac is a prodrug that is deaminated to its active metabolite (amfenac) in the ocular tissues. It is a potent inhibitor of the cyclooxygenase (COX) isoforms COX-1 and COX-2 and is distributed rapidly in both the anterior and posterior segments of the eye. It is well known that the retinal thickness increase is significantly lower in patients administered an NSAID after cataract surgery [23, 24]. It may therefore be better to avoid NSAID use when evaluating retinal layer thickness after cataract surgery.

Our study has a few limitations. First, the sample size could be larger. Second, the retinal thickness values continued to show a slight decrease at the sixth month visit, and the follow-up should therefore be longer than 6 months.

In conclusion, we presented the six-month follow-up results of TRT, TRV, and retinal layer thickness after uncomplicated cataract surgery in this study. The thickest values were observed at the first and third month visits. A slight decrease without reaching preoperative levels was found in all thickness parameters at the sixth month visit. The postoperative thickness increase was more prominent in the 1–3 mm circle than in the 1 mm circle. On the other hand, OPL was the only retinal layer with decreased thickness after surgery. These findings may be useful for understanding the pathophysiological pathways of PCME. The results suggest that long-term follow-up of more than 6 months is needed to see whether total retinal and segmental changes return to preoperative levels.

## Figures and Tables

**Table 1:** Thickness of macula TRT, TRV, and retinal layers with at the ETDRS circle of 1 and 3 millimeters.

		Preoperative	Postoperative day 1	Postoperative week 1	Postoperative month 1	Postoperative month 3	Postoperative month 6	*p*
TRT	1 mm circle	276.63 ± 27.36	272.14 ± 26.12^*∗*^	274.85 ± 26.74	279.81 ± 25.80	280.65 ± 26.82^*∗*^	277.85 ± 26.22	<0.001
	3 mm circle	332.25 ± 14.66	327.27 ± 13.78^*∗*^	332.81 ± 13.99	337.31 ± 13.21^*∗*^	337.95 ± 14.24^*∗*^	337.01 ± 14.59^*∗*^	<0.001

TRV	1 mm circle	0.2167 ± 0.0215	0.2143 ± 0.0201	0.2167 ± 0.0203	0.2205 ± 0.0201^*∗*^	0.2214 ± 0.0208^*∗*^	0.2186 ± 0.0206	<0.001
	3 mm circle	0.5237 ± 0.0241	0.5170 ± 0.0240^*∗*^	0.5252 ± 0.0231	0.5315 ± 0.0224^*∗*^	0.5332 ± 0.0238^*∗*^	0.5315 ± 0.0246^*∗*^	<0.001

RPE	1 mm circle	15.15 ± 2.20	14.70 ± 1.72	15.30 ± 1.97	15.25 ± 1.86	15.20 ± 2.14	15.45 ± 2.03	0.453
	3 mm circle	14.26 ± 1.61	13.75 ± 1.81	14.02 ± 1.60	14.40 ± 1.72	14.03 ± 1.94	14.13 ± 1.39	0.042^*∗∗*^

ONL	1 mm circle	88.32 ± 13.96	86.26 ± 14.29	91.58 ± 10.09	91.05 ± 14.20	92.26 ± 12.55	91.32 ± 13.83	<0.001^*∗∗∗*^
	3 mm circle	70.13 ± 7.83	68.86 ± 7.60	71.68 ± 6.74	72.63 ± 7.87	73.85 ± 7.88^*∗*^	73.31 ± 7.58^*∗*^	<0.001

OPL	1 mm circle	26.26 ± 7.10	26.58 ± 6.00	23.63 ± 4.87	26.05 ± 6.38	25.74 ± 5.07	25.37 ± 6.31	0.144
	3 mm circle	32.11 ± 2.67	31.07 ± 3.07	29.51 ± 2.51^*∗*^	30.20 ± 2.59	30.23 ± 2.86	29.64 ± 2.27^*∗*^	0.001

INL	1 mm circle	24.16 ± 8.20	24.21 ± 9.63	23.42 ± 8.60	24.16 ± 8.40	24.21 ± 8.03	24.26 ± 9.36	0.881
	3 mm circle	40.64 ± 3.71	40.27 ± 4.33^*∗*^	41.56 ± 4.10^*∗*^	41.55 ± 4.63^*∗*^	41.94 ± 4.52^*∗*^	42.36 ± 4.73^*∗*^	<0.001

IPL	1 mm circle	23.37 ± 7.41	22.26 ± 6.40	22.47 ± 7.47	23.26 ± 7.26	23.16 ± 7.82	23.47 ± 7.31	0.055
	3 mm circle	39.94 ± 3.23	39.63 ± 3.05	40.63 ± 3.41	41.05 ± 3.49^*∗*^	41.05 ± 3.51^*∗*^	41.48 ± 3.83^*∗*^	<0.001

GCL	1 mm circle	18.58 ± 10.65	18.32 ± 10.84	18.95 ± 10.63	19.00 ± 11.23	18.95 ± 10.12	18.63 ± 11.10	0.298
	3 mm circle	47.98 ± 5.42	47.55 ± 5.30	48.93 ± 5.21	49.61 ± 5.26^*∗*^	49.88 ± 5.40^*∗*^	49.65 ± 5.36^*∗*^	<0.001

NFL	1 mm circle	13.20 ± 2.82	13.35 ± 3.45	13.40 ± 3.53	13.45 ± 3.80	14.05 ± 4.38	13.20 ± 3.31	0.525
	3 mm circle	21.96 ± 1.62	22.13 ± 1.87	22.69 ± 1.66^*∗*^	23.02 ± 1.82^*∗*^	22.96 ± 1.77^*∗*^	22.59 ± 1.50	<0.001

TRT, total retinal thickness; TRV, total retinal volume; RPE, retinal pigment epithelium; ONL, outer nuclear layer; OPL, outer plexiform layer; INL, inner nuclear layer; IPL, inner plexiform layer; GCL, ganglion cell layer; RNFL, retinal nerve fiber layer. ^*∗*^Difference with preoperative measurement statistically significant using the Bonferroni correction. ^*∗∗*^The differences between the mean RPE values were significant according to repeated measure results, but the Bonferroni test did not reveal a significant change. ^*∗∗∗*^The Bonferroni test did not reveal a significant change between the preoperative and postoperative mean ONL values. However, the decrease in the mean value in the postoperative first day has resulted in a significant difference between the mean first day value and the mean 3rd month value with Bonferroni correction.

**Table 2:** The effect of EPT and TE on GCL and INL.

ETDRS circle		1st day	1st week	1st month	3rd month	6th month
1 mm circle	EPT versus GCL	*p*=0.021*r*=0.511	*p*=0.039*r*=0.443	*p*=0.038*r*=0.467	*p*=0.034*r*=0.501	*p*=0.076*r*=0.406
	TE versus GCL	*p*=0.026*r*=0.495	*p*=0.047*r*=0.427	*p*=0.025*r*=0.499	*p*=0.025*r*=0.525	*p*=0.078*r*=0.403

3 mm circle	EPT versus INL	*p*=0.027*r*=0.494	*p*=0.044*r*=0.433	*p*=0.014*r*=0.538	*p*=0.084*r*=0.418	*p*=0.025*r*=0.500
	TE versus INL	*p*=0.004*r*=0.614	*p*=0.011*r*=0.528	*p*=0.003*r*=0.634	*p*=0.027*r*=0.520	*p*=0.003*r*=0.627

EPT, effective phaco time; GCL, ganglion cell layer; TE, total energy; INL, inner nuclear layer.

## Data Availability

The data used to support the findings of this study are available from the corresponding author upon request.

## References

[1] Kessel L., Tendal B., Jørgensen K. J. (2014). Post-cataract prevention of inflammation and macular edema by steroid and nonsteroidal anti-inflammatory eye drops: a systematic review. *Ophthalmology*.

[2] Yonekawa Y., Kim I. K. (2012). Pseudophakic cystoid macular edema. *Current Opinion in Ophthalmology*.

[3] Lobo C. (2012). Pseudophakic cystoid macular edema. *Ophthalmologica*.

[4] Ctori I., Huntjens B. (2015). Repeatability of foveal measurements using spectralis optical coherence tomography segmentation software. *PLoS One*.

[5] Yilmaz T., Cordero-Coma M., Gallagher M. J. (2012). Ketorolac therapy for the prevention of acute pseudophakic cystoid macular edema: a systematic review. *Eye*.

[6] Salomon L. D. (1995). Efficacy of topical flurbiprofen and indomethacin in preventing pseudophakic cystoid macular edema. Flurbiprofen—CME study group I. *Journal of Cataract and Refractive Surgery*.

[7] Grewing R., Becker H. (2000). Retinal thickness immediately after cataract surgery measured by optical coherence tomography. *Ophthalmic Surgery and lasers*.

[8] Perente I., Utine C. A., Ozturker C. (2007). Evaluation of macular changes after uncomplicated phacoemulsification surgery by optical coherence tomography. *Current Eye Research*.

[9] Šiško K., Knez N. K., Pahor D. (2015). Influence of cataract surgery on macular thickness: a 6-month follow-up. *Wiener klinische Wochenschrift*.

[10] Gharbiya M., Cruciani F., Cuozzo G., Parisi F., Russo P., Abdolrahimzadeh S. (2013). Macular thickness changes evaluated with spectral domain optical coherence tomography after uncomplicated phacoemulsification. *Eye*.

[11] Falcão M. S., Gonçalves N. M., Freitas-Costa P. (2014). Choroidal and macular thickness changes induced by cataract surgery. *Clinical Ophthalmology*.

[12] Cagini C., Fiore T., Iaccheri B., Piccinelli F., Ricci M. A., Fruttini D. (2009). Macular thickness measured by optical coherence tomography in a healthy population before and after uncomplicated cataract phacoemulsification surgery. *Current Eye Research*.

[13] Nicholas S., Riley A., Patel H., Neveldson B., Purdie G., Wells A. P. (2006). Correlations between optical coherence tomography measurement of macular thickness and visual acuity after cataract extraction. *Clinical and Experimental Ophthalmology*.

[14] Von Jagow B., Ohrloff C., Kohnen T. (2007). Macular thickness after uneventful cataract surgery determined by optical coherence tomography. *Graefe’s Archive for Clinical and Experimental Ophthalmology*.

[15] Biro Z., Balla Z., Kovacs B. (2008). Change of foveal and perifoveal thickness measured by OCT after phacoemulsification and IOL implantation. *Eye*.

[16] Kusbeci T., Eryigit L., Yavas G., Inan U. U. (2012). Evaluation of cystoid macular edema using optical coherence tomography and fundus fluorescein angiography after uncomplicated phacoemulsification surgery. *Current Eye Research*.

[17] Gołebiewska J., Kęcik D., Turczyńska M., Moneta-Wielgoś J., Kopacz D., Pihowicz-Bakoń K. (2014). Evaluation of macular thickness after uneventful phacoemulsification in selected patient populations using optical coherence tomography. *Klinika Oczna*.

[18] Park H. Y., Kim J. H., Park C. K. (2014). Neuronal cell death in the inner retina and the influence of vascular endothelial growth factor inhibition in a diabetic rat model. *American Journal of Pathology*.

[19] Sigler E. J., Randolph J. C., Kiernan D. F. (2016). Longitudinal analysis of the structural pattern of pseudophakic cystoid macular edema using multimodal imaging. *Graefe’s Archive for Clinical and Experimental Ophthalmology*.

[20] Shaik-Dasthagirisaheb Y. B., Varvara G., Murmura G. (2013). Vascular endothelial growth factor (VEGF), mast cells and inflammation. *International Journal of Immunopathology and Pharmacology*.

[21] Kaushik M., Wang C. Y., Barnett M. H. (2013). Inner nuclear layer thickening is inversely proportional to retinal ganglion cell loss in optic neuritis. *PLoS One*.

[22] Knier B., Schmidt P., Aly L. (2016). Retinal inner nuclear layer volume reflects response to immunotherapy in multiple sclerosis. *Brain*.

[23] Singh R. P., Staurenghi G., Pollack A. (2017). Efficacy of nepafenac ophthalmic suspension 0.1% in improving clinical outcomes following cataract surgery in patients with diabetes: an analysis of two randomized studies. *Clinical Ophthalmology*.

[24] Chastain J. E., Sanders M. E., Curtis M. A. (2016). Distribution of topical ocular nepafenac and its active metabolite amfenac to the posterior segment of the eye. *Experimental Eye Research*.

